# The complete chloroplast genome sequence of *Artabotrys pilosus* (Annonaceae)

**DOI:** 10.1080/23802359.2021.2005495

**Published:** 2021-12-10

**Authors:** Ze-rui Liang, Jin-man Lei, Hui Zhang, Yong-quan Li, Bipei Zhang

**Affiliations:** College of Horticulture and Landscape Architecture, Zhongkai University of Agriculture and Engineering, Guangzhou, China

**Keywords:** Annonaceae, *Artabotrys pilosus*, complete chloroplast genome, phylogenetic analysis

## Abstract

*Artabotrys pilosus* (Annonaceae) is endemic to China, this plant has high medicinal value and broad application prospect. In this study, we assembled and systematically analyzed the chloroplast genome of *A. pilosus* on the basis of DNA sequencing using high-throughput techniques. The chloroplast sequence of *A. pilosus* was 178,195 bp in length, including two inverted repeat regions of 42,150 bp, a large single-copy region of 90,797 bp and a small single-copy region of 3098 bp. It was predicted to contain 142 genes, of which 96 are coding, 38 are tRNA genes, and eight are rRNA genes. The overall GC content was 38.8%; this was higher in the IRs (40.4%) when compared to the LSC (37.6%) and the SSC (32%) regions. Phylogenetic analysis showed that *A. pilosus* is in subfamily Annonoideae.

Annonaceae is a diverse family with *ca.* 107 genera and 2400 species of trees, shrubs, and lianas (Guo et al. 2017). *Artabotrys* R.Br. is one of the largest genera in Annonaceae with over 100 species distributed in Africa and Asia (Chen and Eiadthong 2020; Xue et al. 2021). Many species in this genus have been used as a traditional folk medicinal plant for the treatment of malaria, lymphatic tuberculosis, and is rich in anti-tumor active ingredients, and have received extensive attention (e.g. Murphy et al. 2008; Zhou et al. 2015; Mehta et al. 2017). *Artabotrys pilosus* Merr. & Chun 1935 is an endemic species in China distributed in Hainan and Guangdong provinces (Tsiang and Li 1979; Li and Gilbert 2011). Some studies revealed that the constituents in the extracts from *A. pilosus* exhibited inhibitory activities toward a number of human cancer cell lines, such as HL-60, A549, SW480, etc. (Liu et al. 2015; Wang 2016). However, researches on chloroplast genome of *A. pilosus* have not been reported. In this study, we sequence the chloroplast complete genome of *A. pilosus*, which represent the first plastid genome in *Artabotrys*. The data would be helpful for the phylogenetic study of the large genus and related genera in the future.

The fresh leaves of *A. pilosus* were collected from South China Botanical Garden, Guangzhou, China (23°11′4.7′′N, 113°21′49.1′′E). The voucher specimen (*B. Xue 316*) was deposited in the herbaria of the South China Botanical Garden, Chinese Academy of Sciences (IBSC, http://herbarium.scbg.cas.cn/, Feiyan Zeng, zengfeiy@scbg.ac.cn). Total geonomic DNA of *A. pilosus* was extracted using the modified CTAB method (Doyle and Doyle 1987). Library construction and sequencing were performed by BGI-Shenzhen (Shenzhen, China), using an Illumina HisSeq 2500 Sequencing System following manufacturer’s instructions. Plastid sequence reads were assembled using the software NOVOPlasty (Dierckxsens et al. 2017). The genome was then annotated by PGA (Qu et al. 2019). The annotation results were than manually corrected in the software Geneious 9.0.2 (Kearse et al. 2012). Finally, the complete sequences and annotations of *A. pilosus* were submitted to GenBank with the accession number OK216144.

The length of *A. pilosus* complete chloroplast genome sequence was 178,195 bp, with a large single-copy (LSC) region of 90,797 bp, a small single-copy (SSC) region of 3,089 bp, and two inverted repeat (IR) regions of 42,150 bp each. The overall GC content was 38.8%, the LSC, SSC, and IR regions GC content was 32.0%, 37.6%, and 40.4% respectively. A total of 142 genes were predicted, including 96 protein-coding genes, 38 tRNA genes, and eight rRNA genes. Compared with the plastid genome of *Polyalthiopsis verrucipes* (159,960 bp, MW018366), the plastid genome of *Artabotrys pilosus* newly sequenced here has much shorter SSC and longer IR regions, with nine genes (i.e. *ndhA*, *ndhD*, *ndhE*, *ndhG*, *ndhH*, *ndhI*, *psaC*, *rps15*, *trnL-UAG*) expanded to IR regions. The expanded IR regions is also reported in several other Annonaceae species, such as *Annona reticulata* (201,906bp, NC052009) and *Uvaria macrophylla* (192,782bp, NC041442).

To investigate the phylogenetic position of *Artabotrys pilosus*, an ML tree was constructed for *A. pilosus* and eight other representative species from Annonaceae, with four species from Magnoliaceae and Myristicaceae as outgroups. The complete chloroplast genome sequences of the above-mentioned 13 species were aligned using MAFFT v7.307 (Katoh and Standley 2013). A maximum likelihood analysis was performed by RAxML (Stamatakis 2014) provided by CIPRESScience Gateway (Miller et al. 2015) under GTR + G model with 1000 bootstrap replicates.

As shown in Figure 1, A. *pilosus* is in the subfamily Annonoideae in Annonaceae. The phylogenetic relationships of the four genera with available plastid genomes in subfamily Annonoideae, i.e. (((*Fissistigma*-*Uvaria*)-*Annona*)-*Artabotrys*), is consistent with previous phylogenetic results based on several plastid regions (Guo et al. 2017; Xue et al. 2020).

**Figure 1.**
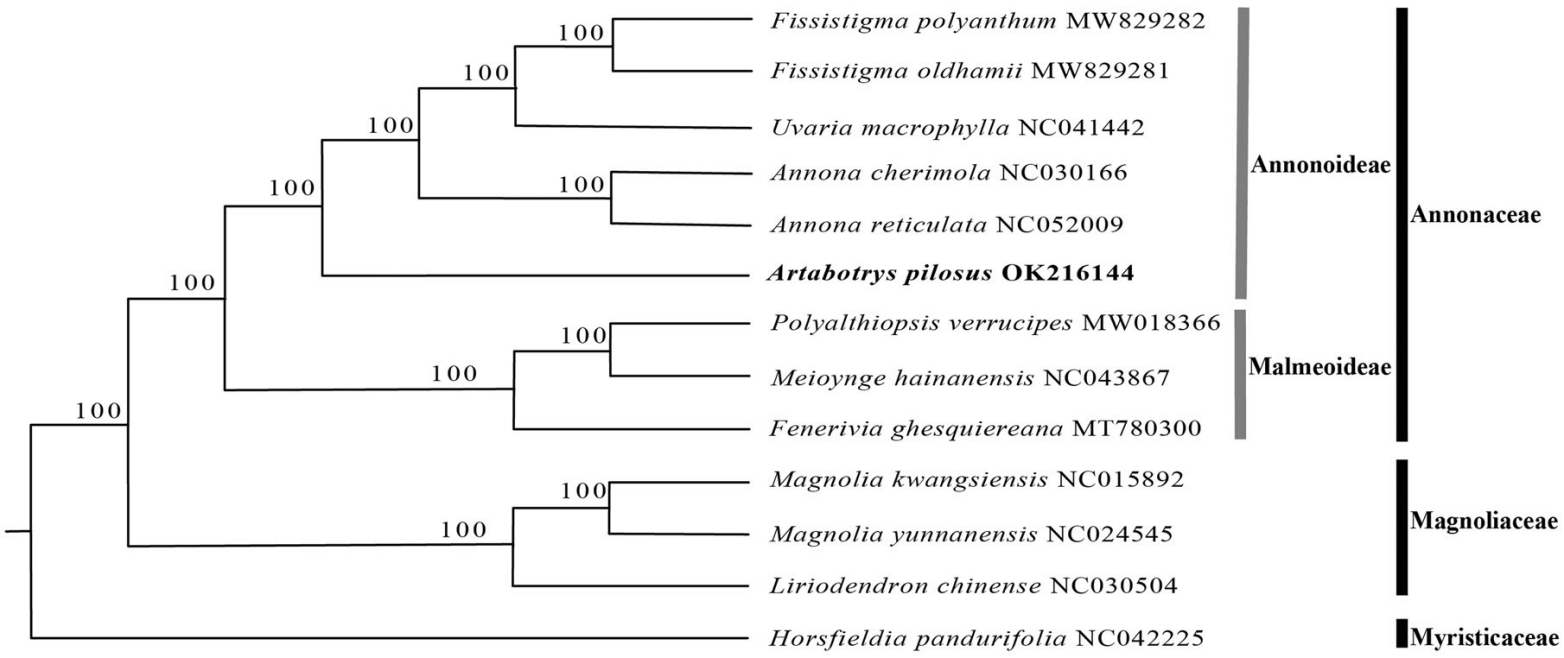
Maximum likelihood tree of *A. pilosus* and related species based on whole chloroplast genome sequences. Numbers beside each node are bootstrap values.

## Data Availability

The genome sequence data that obtained at this study are openly available in GenBank of NCBI (https://www.ncbi.nlm.nih.gov/) under the accession number of OK216144. The associated BioProject, Bio-Sample and SRA, numbers are PRJNA764536, SAMN21508033 and SRR16018935, respectively.
